# miR-497 expression, function and clinical application in cancer

**DOI:** 10.18632/oncotarget.10152

**Published:** 2016-06-17

**Authors:** Gang Yang, Guangbing Xiong, Zhe Cao, Suli Zheng, Lei You, Taiping Zhang, Yupei Zhao

**Affiliations:** ^1^ Department of General Surgery, Peking Union Medical College Hospital, Chinese Academy of Medical Sciences and Peking Union Medical College, Beijing, China

**Keywords:** miR-497, carcinogenesis, biomarker, clinical application

## Abstract

MicroRNAs (miRNAs) are small non-coding RNAs that inhibit gene expression by binding to the 3′ untranslated region (3′-UTR) of their target mRNAs. Recent studies show that miR-497 plays an important role in various cancers. Here, we summarize the existing studies of miR-497 as following: (1) miR-497 expression in cancer; (2) regulation mechanisms of miR-497 expression; (3) function of miR-497 in cancer; (4) direct targets of miR-497; (5) Clinical applications of miR-497. Recent analyses verify that miR-497 mainly suppresses tumors; however, it also acts as an oncogene in several cancers. Increasing evidence indicates that miR-497 can serve as a diagnostic and prognostic biomarker and is a promising therapeutic target for future clinical applications.

## INTRODUCTION

MicroRNAs (miRNAs) comprise a class of small non-coding RNAs of 19-25 nucleotides [1]. They are transcribed as a primary miRNA (pri-miRNA) and processed in the nucleus by Drosha to form an ~70-nt precursor miRNA (pre-miRNA) [2]. Then, the pre-miRNA is transported to the cytoplasm by exportin-5 and further processed by Dicer and TRBP2 to generate an imperfect mature miRNA: miRNA* duplex [3–5]. After the miRNA duplex is separated, the mature miRNA molecule is incorporated into the RNA-induced silenced complex (RISC) where it binds to a member of the Argonaute (AGO) protein family. The other transient strand, miRNA*, is usually degraded by AGO2 [6, 7]. The active RISC complex then binds to an mRNA transcript with a complementary sequence to inhibit it at the post-transcriptional level [8]. miRNA dysregulation is involved in the initiation and progression of various diseases [9].

miR-497, a highly conserved miRNA encoded by the first intron of the MIR497HG (Gene ID: 100506755) gene on human chromosome 17p13.1 [10], belongs to the miR-15 family (miR-15a, miR-15b, miR-16-1/2, miR-195, miR-424 and miR-497), sharing the same 3′-UTR binding seed sequence AGCAGCA [11]. The characteristics of some miR-15 family members, including miR-15, miR-16 [12] and miR-195 [13], have been summarized elsewhere; therefore, we focused on the most recent findings regarding miR-497. miR-497 is found in almost all human organs and tissues, including the brain [14], breast [15], lung [16], kidney [17], stomach [18], liver [9] and blood [19]. Several studies have confirmed its involvement in human disease. To better understand the role of miR-497 in cancers, we summarize recent studies investigating the expression, regulatory, biological functions, targets and clinical value of miR-497. Our goal is to provide useful information for miR-497 future clinical applications.

## EXPRESSION OF MIR-497 IN CANCER

The first indication of miR-497 dysregulation was based on an investigation of global miRNA expression profiles in primary breast cancer [20]. Decreased miR-497 expression in breast cancer was verified in subsequent studies [15, 21–23]. A recent study that profiled miRNAs in male breast cancer found that miR-497 was among the most prominently down-regulated miRNAs [24]. Pinto et al. observed that in familial breast cancer, miR-497 was statistically significantly over-expressed in males (*n* = 23, *p* = 0.008) compared with females [25]. miR-497 down-regulation has also been consistently demonstrated in a variety of other tumor types, including gastric cancer (GC) [26], colorectal cancer (CRC) [27], hepatocellular carcinoma (HCC) [28], pancreatic cancer [29], adrenocortical carcinoma (ACC) [30], bladder cancer [19], non-small cell lung cancer (NSCLC) [16], melanoma [31], ovarian cancer (OC) [32], and cervical cancer (CC) [33] and other solid tumors. These results suggest that miR-497 has a tumor-suppressive role. miR-497 expression levels in most cell lines of these types of cancer are also decreased. (The specific expression of miR-497 in cancers is shown in Table 1.)

Although Jiang et al. reported that the miR-497 level was increased in 25 CRC tumor tissues compared with adjacent normal tissues from the same patient [34], Guo et al. [35] confirmed miR-497 down-regulation in 107 of 137 paired samples from 10 CRC patients. Wang et al. found significantly lower miR-497 expression levels in CRC tumor tissues than in adjacent normal tissues [36]. We speculate that this discrepancy regarding miR-497 expression may be related to technical variations in the miRNA isolation method or different patient states, such as before or after chemotherapy. For example, Qiu et al. revealed that bufalin treatment could increase the miR-497 level in HCT116 CRC cells [37]. Nonetheless, in a study of 184 CC patients, 186 cervical intraepithelial neoplasia (CIN) patients and 193 healthy controls [38], serum miR-497 was significantly increased (P < 0.001) in CC patients, which is inconsistent with the down-regulation of miR-497 in tumor tissues [33]. Thus, it is possible that tumor cells are not a major contributor to elevated circulating miR-497 levels. Further studies should explore the potential effects of disparate miR-497 expression on CC development.

More recent studies have shown that miR-497 is up-regulated in several other cancers. In the leukemic cells of subset #4 cases, miRNAs expression profiling revealed significant up-regulation of in CD19+ cells compared with the normal B-cell population in chronic lymphocytic leukemia (CLL) [39]. miR-497 is also over-expressed in glioma [40] and diffuse large B-cell lymphoma (DLBCL) [41]. These findings indicate that miR-497 may have different roles in tumorigenesis.

## REGULATION MECHANISMS OF MIR-497 EXPRESSION

### Transcription factors

Transcription factors (TFs) mainly bind to specific DNA sequences through multiple mechanisms, either promoting or repressing miRNA transcription [42].

**Figure 1:**
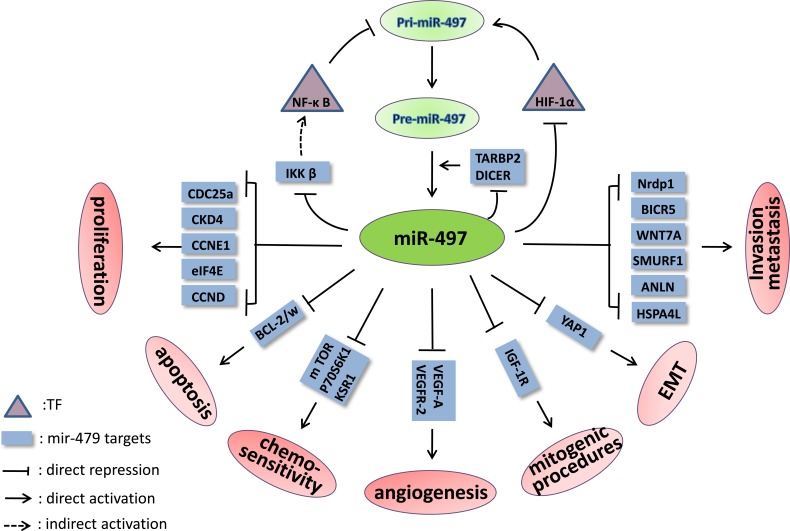
The signaling pathways and feedback loops in which miR-497 is involved The feedback loops of miR-497-HIF-1α-*miR-497*, mir-497-IKKβ-IκBs-NF-κB-*miR-497* and miR-497-TARBP2/DICER-miR-497 are shown.

Hypoxia-inducible factor-1α (HIF-1α) is a key TF induced by hypoxia and can increase the expression of many genes [43, 44]. Although miR-497 expression is decreased in retinoblastoma cells under hypoxic conditions [45], Lan et al. discovered that hypoxia and DOMG, a well-established agent that induces HIF-1α, significantly increased pri-miR-497 levels [40]. The authors also used chromatin immunoprecipitation (ChIP) assays to demonstrate that HIF-1α binds to HRE1 elements. The hypoxia-induced increase in luciferase activity was significantly inhibited with a mutated HRE1 in the core binding site; this eliminated HIF-1α binding and indicated that HIF-1α activates miR-497 transcription by directly binding to the HRE1 element upstream of the promoter. Wu et al. confirmed that miR-497 over-expression could decrease the hypoxia-induced increase in HIF-1α protein levels [46]. Therefore, miR-497 and HIF-1α form a negative feedback loop, as Figure 1 shows.

Nuclear factor-kappa B (NF-κB), another TF, participates in various biological processes with pleiotropic activity and in the expression of many genes and miRNAs when present in the nucleus in its active form [47, 48]. Wei et al. observed that p65 (also known as Rel A, the active subunit of NF-κB) can directly bind to a specific miR-497 promoter site in C2C12 myoblast cells and reduce its expression, as determined by luciferase and ChIP-PCR assays [49]. However, Mechtler et al. reported that PIPK-1 and IL-1β, which activate NF-κB, can increase the miR-497 level [50], though the direct relationship between miR-497 and NF-κB and the miR-497 gene NF-κB binding site was not exhaustively described. Hence, we speculate that the effect of PIPK-1 and IL-1β on miR-497 expression may occur in an NF-κB-independent manner.

### Epigenetic alterations

Similar to protein-coding genes, miRNAs undergo epigenetic modulation [51].

Indeed, DNA methylation at CpG dinucleotides can affect miRNAs expression [52]. For example, Wiklund et al. found that miR-497 was silenced concomitantly with DNA hypermethylation of CpGs in the upstream region of the miR-497 promoter in breast cancer tissues and cells [15]. Furthermore, Cai et al. reported that betaine, a methyl provider that can modulate gene expression through DNA methylation [53–56], led to a significant reduction in hepatic miR-497 expression in newborn piglets when administered to their mothers during gestation. This finding indicates a strong association between DNA methylation and miR-497 levels [57]. Additionally, Menigatti et al. showed that treatment with the DNA-demethylating agent 5-aza-2-deoxy-cytidine (5-Aza-dC) could restore miR-497 expression, which is suppressed in HT29 CRC cells [27]. Furthermore, the CpG island upstream of the transcription start site (TSS) for was found to be monoallelically methylated in 50/50 samples from normal colorectal mucosa but fully methylated in 38/50 (76%) samples from colorectal adenomas. Additionally, miR-497 expression decreased significantly in the colorectal adenomas, suggesting that the *miR-497* locus might be methylation imprinted during colorectal tumorigenesis. Recently, miR-497 was also found to be down-regulated in HCC [58] and OC [59] as a result of hypermethylation of the promoter region.

Although these studies have established that DNA methylation regulates miR-497, other regulatory mechanism of epigenetic alterations, such as histone modification and genomic imprinting have not been reported yet. More research is needed.

### Genomic alterations

In addition to TFs and epigenetic modification, genomic alterations have also been reported to control miR-497 expression in cancer cells. For example, using array comparative genomic hybridization (aCGH), Guo et al. found that the segment of chromosome 17p13.1 that harbors miR-497 is deleted in colon cancer samples with lower miR-497 expression compared with normal mucosa [35]. In a large cohort of 131 paired CRC tissues, ~71% of colon cancers exhibited DNA copy number reduction at this segment. The fact that colon cancer samples lacking this fragment have lower miR-497 levels than cancer samples that retain the fragment indicates that the observed gene copy number reduction is directly responsible for miR-497 decrease. Consistently, Flavin et al. reported that miR-497 down-regulation may be caused by distinct allelic loss patterns in peritoneal carcinoma cells [60], whereas Vaishnavi et al. found that miR-497 up-regulation in autism is correlated with a duplicated copy number variation (CNV) locus [61].

Furthermore, recent studies reported that long non-coding RNAs (lncRNAs), which can binding and sequester miRNAs and act as so-called miRNAs sponges, could inhibit miR-497 expression [62, 63]. However, no study to date has reported a relationship between lncRNAs and miR-497.

## FUNCTION OF MIR-497 IN CANCER

Both gain- and loss-of-function approaches are commonly utilized to examine miR-497 function. The following studies based on these methods support an anti-oncogenic role for miR-497. Several studies have reported that increasing miR-497 expression *via* pre-miR-497 transfection suppresses proliferation and increases apoptosis in ACC [30] and pancreatic cancer [64] and inhibits migration and invasion in bladder cancer [10] and nasopharyngeal carcinoma [65]. Additionally, miR-497 over-expression was found to block G0/G1 phase transition in MCF-7 breast cancer cells [66] and induce G1/S arrest in SGC-7901 GC cells [26]. miR-497 up-regulation by lentivirus LV-miR-497 transfection also significantly represses tumor angiogenesis in HCC [67] and OC [68]. Conversely, the inhibition of miR-497 expression by an miR-497 inhibitor increased the growth and colony formation ability of breast cancer cells but reduced their apoptosis [69]. Moreover, decreases in miR-497 led to increased migration and invasion capacities in prostate cancer cells [70], proliferation in NSCLC [71] and chemoresistance in osteosarcomas [72]. Our lab found that in pancreatic cancer, enhanced miR-497 could suppress cell proliferation, induce cycle arrest, attenuate migration and invasion capacities and inhibit tumor growth *in vivo* [29, 64].

In addition to a tumor suppressor role, miR-497 may also act as an oncogene. Lan et al. discovered that miR-497 over-expression protects tumor cells from apoptosis and enhances resistance to temozolomide in glioma [40]. Interestingly, when miR-497 expression was increased, the ability to metastasize was abolished in HCT116 CRC cells [35] but enhanced in Caco-2 CRC cells [34]. Such results may be explained by differences in the cell lines.

Therefore, miR-497 largely functions as a tumor suppressor in cancer, but it also acts as an oncogene by inhibiting or activating multiple biological processes, including division, proliferation, apoptosis, angiogenesis, migration, and invasion.

## DIRECT TARGETS OF MIR-497

miRNAs inhibit target gene expression primarily by binding to the 3′-UTRs of target mRNAs, leading to mRNA cleavage or translational repression [73, 74]. Some miR-497 targets that have been fully validated are discussed below.

B cell lymphoma w (Bcl-w) and Bcl-2 are members of the Bcl-2 family and key regulators of cell apoptosis [75]. They are the primary miR-497 targets. Lei et al. found that Bcl-w reduced apoptosis in tumor cells and that miR-497 could significantly inhibit its expression at both the mRNA and protein levels in breast cancer [66]. Yin et al. used a luciferase reporter assay to demonstrate that miR-497 could directly bind to the 3′-UTR of Bcl-w [76]. In addition, Bcl-2, an anti-apoptosis factor that contributes to tumorigenesis and chemoresistance, was found to be a target of miR-497 in breast cancer [69], GC [77] and CLL [39]. Bcl-w and Bcl-2 also promote the migration and invasion of cells by stimulating downstream factors, including phosphoinositide 3-kinase (PI3K)/ AKT, epidermal growth factor receptor (EGFR), matrix metalloproteinase (MMP)-2, and urokinase-type plasminogen activator (uPA) [78]. Clearly, Bcl-w and Bcl-2 are involved in the anti-cancer effect of miR-497.

Vascular endothelial growth factor A (VEGF-A) is the most potent angiogenic factor. It is responsible for angiogenesis *via* a VEGF receptor 2 (VEGFR-2)-dependent signaling pathway [79] and is important for tumor growth, invasiveness and metastasis [80].miR-497 regulation of VEGF-A expression has been confirmed in several cancers. Recent studies have revealed that VEGF-A is a critical target that controls miR-497 repression of tumor growth and invasion in non-small lung cancer [71] and angiogenesis and metastasis in HCC [67]. Shao et al. revealed that VEGF-A over-expression induced by miR-497 inhibitors increased the protein level of phospho-AKT, which subsequently up-regulates Bcl- 2 and Cyclin D 1 (CCND1) protein and promoted cell growth in SAOS-2 osteosarcoma cells [72]. Additionally, Wang et al. demonstrated that levels of AKT and extracellular signal-regulated kinase 1/2 (ERK1/2), signaling molecules downstream of VEGF-A, are significantly decreased in miR-497-transfected OC cells, which inhibits angiogenesis [68]. Interestingly, Tu et al. discovered that miR-497 also targets VEGFR-2 to inhibit HUVEC apoptosis and tumor angiogenesis in breast cancer [81]. These results suggest that miR-497 may inhibit angiogenesis and tumor growth by reducing VEGF-A and VEGFR-2 expression through PI3K/AKT and mitogen-activated protein kinase (MAPK)/ERK pathways. Therefore, VEGF-A and VEGFR-2 may be key mediators of the miR-497 anti-angiogenesis effect on tumor progression.

Insulin-like growth factor receptor 1 (IGF-1R), a transmembrane tyrosine kinase, can activate multiple signaling pathways to control mitogenic procedures when bound to a ligand [82–84]. Recent studies showed that miR-497 targeted IGF-1R to inhibit proliferation, invasion and metastasis by regulating PI3K/AKT signaling pathway activation in colorectal cancer [35] and to function as an oncogene in cervical cancer [33]. Additionally, our lab demonstrated that suppressed IGF-1R protein expression *via* miR-497 up-regulation was able to re-sensitize pancreatic cancer cells to gemcitabine and that plasma IGF-1R can discriminate pancreatic cancer from other pancreatic tumors [64]. Furthermore, insulin receptor (IR), which mediates similar downstream signaling pathways as IGF-1R, has also been verified as an miR-497 target, and it plays an important role in myogenesis [49] and metabolic regulation [85]. Such studies indicate that miR-497-IGF-1R/IR is likely a clinically significant tumor suppressor-oncogene pair in human carcinomas and a critical mediator of normal biological functions.

Cyclin E1 (CCNE1) is a member of the cyclin family. It binds to and activates cyclin-dependent kinase 2 (CDK2) to promote cell cycle progression from the G1 to the S phase by initiating a cascade of events [86, 87]. Over-expression of CCNE1 has been detected in various cancers [88], and recent studies have revealed that miR-497 can directly reduce the CCNE1 protein level to suppress tumor growth by inducing G1 arrest in breast cancer [22] and HCC [89]. Han et al. discovered that when CCNE1 was knocked down, the inhibitory effect on cell proliferation was not enhanced by miR-497 over-expression in lung cancer, indicating that CCNE1 mediates the effects of miR-497 on cell growth [90]. Several other genes encoding cell cycle activators, including cyclins [15, 91], cyclin-dependent kinase 4 (CDK4) [89], eukaryotic translation initiation factor 4E (eIF4E) [26] and cell cycle division factors (CDC25a) [92], are also known targets of miR-497.

Inhibitors of NF-κ B kinase β (IKKβ), which is the key kinase in the canonical NF-κ B signaling pathway, is involved in cell proliferation, metastasis, invasion and the epithelial-mesenchymal transition (EMT) [93]. IKKβ can promote the translocation of cyto­plasmic NF-κB into the nucleus by phosphorylating inhibitors of NF-κB (IκBs), thus increasing the expression of genes involved in cancer progression [94]. Mechtler et al. used a luciferase reporter assay to confirm that IKKβ mRNA 3′-UTR harbors a miR-497 binding site, and miR-497 transfection into C6TA4 cells resulted in a notable reduction in IKKβ protein level [50]. In prostate cancer, the suppression of IKKβ/CDK8/MMP-9 signaling *via* miR-497 over-expression led to nearly 50% inhibition of PC3-AR cell migra­tion and invasion [95]. As described above, NF-κB directly inhibits miR-497 transcription [49]. Therefore, miR-497 and the NF-κB pathway form a mutually inhibitory miR-497-IKKβ-IκBs-NF-κB-miR-497 feedback loop. (Figure 1)

TARBP2 and DICER play central roles in the miRNAs process by cleaving pre-miRNAs to form mature miRNAs. However, Caramuta et al. reported that miR-497 could down-regulate these factors in NCI-H295R ACC cells [96]. In this study, significant enrichment of endogenous mRNAs was observed for both genes in immunoprecipitated Ago2 complexes when miR-497 was over-expressed, indicating that they are direct targets of miR-497. Therefore, the relevant negative feedback loop may involve mutual control of miR-497 and TARBP2/DICER.

Several other genes critical to cancer genesis and progression are exclusive targets of miR-497. These genes include Nrdp1 [34], CHEK1 [28], FGF2, FGFR1 [29], BIRC5, WNT7A [10], HDGF [16], MEK1 [97], SMURF1 [98], WEE1 [99], ANLN and HSPA4 L [65], PDCD4 [40], AMOT [100], YAP1 [101], mTOR and p70S6K1 [102]. The miR-497 targets discovered in different cancers are summarized in Table 1.

**Table 1:** The expression, targets, biological functions and role of miR-497 in cancer

cancer type	expression	site (tissue/plasma/cell)	targets	biological functions	role	reference
breast cancer	down	tumor and cell line	Bcl-w,Raf1,Ccnd1,cyclin E1,Bcl-2,VEGF,VEGFR2 and HIF-1α	inhibit angiogenesis, cell cycle, colony formation, proliferation, metastasis and invasion ; increase early apoptosis	tumor suppressor	[15,22, 46, 66, 69, 81]
gastric cancer	down	tumor and cell line	BCL2,eIF4E	inhibit G1/S transition, proliferation, invasion; inhibit tumor growth and metastasis *in vivo*; sensitize anticancer drugs	tumor suppressor	[18,26, 77]
colorectal cancer	down	tumor and cell line	IGF1-R,KSR1	reduce cell proliferation, invasion and migration	tumor suppressor	[35,36]
	up	tumor	Nrdp1	increase cell invasiveness	tumor promoter	[34]
primary peritoneal carcinoma	down	tumor	_	_	_	[60]
hepatocellular carcinoma	down	tumor and cell line	CCNE1,CDC25A,CCND3, CDK4,BTRC,CHEK1,VEGFA and AEG-1	inhibit cell G1/S transition, proliferation, angiogenesis and metastasis; repress microvessel densities, metastasis *in vivo*	tumor suppressor	[28,58, 67, 89]
pancreatic cancer	down	tumor and cell line	IGF-1R,FGF2 and FGFR1	suppress cells proliferation, cycle, migration and invasion; enhance apoptosis and re-sensitize cells	tumor suppressor	[29, 64]
adrenocortical carcinoma	down	tumor	TARBP2 and DICER	reduce cell proliferation; increase apoptosis	tumor suppressor	[30, 96]
renal cell carcinoma	down	tumor and cell line	_	inhibit proliferation, migration and invasion	tumor suppressor	[17]
bladder cancer	down	plasma and tumor	BIRC5 and WNT7A	inhibit proliferation, migration and invasion	tumor suppressor	[10,19]
prostate cancer	down	cell line and serum	IKK beta	inhibit proliferation, migration and invasion; induce apoptosis	tumor suppressor	[70, 95]
lung cancer	down	tumors and cell lines	HDGF, CCNE1, VEGF-A, YAP1	inhibit cell proliferation, invasion and colony formation; inhibit tumor growth *in vivo*	tumor suppressor	[16, 71, 90, 102]
cervical cancer	down	tumor and cell line	IGF-1R,MEK1	suppress migration and invasiveness; induce apoptosis	tumor suppressor	[33, 97]
	up	serum	_	promote apoptosis and inhibit proliferation; suppress tumor growth *in vivo*	tumor suppressor	[38]
ovarian cancer	down	tumor and cell line	VEGF-A, SMURF1, mTOR/P70S6K1	suppress angiogenesis, migration and invasion	tumor suppressor	[59, 68, 98]
retinoblastoma	down	tumor and cell line	WEE1	reduce cell viability and increase apoptosis	tumor suppressor	[45, 99]
the head and neck squamous cell carcinoma	down	tumor and plasma	ANLN and HSPA4L	suppress cell proliferation, migration; induce apoptosis; inhibited tumor growth *in vivo*	tumor suppressor	[65]
glioma	up	tumor and cell line	PDCD4	suppress apoptosis; reduce sensitivity of chemotherapy	tumor promoter	[40]
melanoma	down	tumor and cell line	_	inhibit proliferation and viability	tumor suppressor	[31, 109]
osteosarcoma	down	tumor and cell line	VEGF-A, AMOT	suppress proliferation and invasion; re-sensitize cell	tumor suppressor	[72, 100]
leukemia	up	leukemic cells	BCL2	increase apoptosis	tumor suppressor	[39, 107]

Note: The expression of miR-497 is contradictory in colorectal cancer tissues and discrepant between tumor tissues and serum in cervical cancer.

## CLINICAL APPLICATIONS

### miR-497 as a diagnostic marker

Because miR-497 influences a wide range of tumor processes and is often deregulated in various cancers, it has potential use as a diagnostic biomarker.

Patients with breast cancer exhibit lower miR-497 levels in tumor tissues compared with normal tissues (*p* < 0.0001) [15]. A recent report suggests that miR-497 is significantly up-regulated in typical lung carcinoids compared with atypical lung carcinoids [103]. Additionally, Feinmesser et al. confirmed that a combination of miR-34a and miR-497, both under-expressed in ACC, can discriminate these tumors from adrenocortical adenomas with a sensitivity of 89% and specificity of 100% [104]. Except for in tumor tissues, the circulating miR-497 level can also serve as a biomarker. In general, more advanced or malignant tumors express lower levels of miR-497. For example, miR-497 is negatively correlated with lymph node metastasis and TNM stage in breast cancer [66], and cervical cancer patients with a high FIGO stage have lower miR-497 levels [33].

miR-497 is a widely expressed miRNA in a variety of normal tissues. So its clinical diagnostic worth of miR-497 should be reconsidered, because its level in plasma may be influenced by a lot of physiological or pathological factors besides cancer.

### miR-497 as a prognostic marker

If miR-497 expression is related to cancer progression, its level can be used as a prognostic biomarker. Our lab analyzed data from 90 pancreatic cancers cases and found that a low miR-497 level was an independent negative prognostic factor for survival in pancreatic ductal adenocarcinoma (PDAC) [29]. Furthermore, in a study of 128 breast cancer patients, Wang et al. found that patients with high miR-497 expression had better 5-year disease-free and overall survival compared with patients with low miR-497 expression [106]. Similar findings have been reported in clear cell renal cell carcinoma [17], OC has n

Strikingly, in a national, multicenter, pilot clinical trial, primary plasma cell leukemia patients who failed to respond to frontline therapy showed a significantly higher miR-497 level compared with responders, indicating that miR-497 can also be used to predict curative effects [107].

### Potential therapeutic target

miRNAs have gained increasing attention from researchers worldwide for their known post-transcriptional silencing effects.

In this review, we describe miR-497 as mainly a tumor suppressor gene. These data support an inspiring idea that elevated miR-497 can provide a novel therapeutic approach to inhibiting multiple proteins involved in the formation and progression of cancer. In pancreatic cancer, mice transplanted with SW1990 cells that stably over-expressed miR-497 showed a tumor size reduction of more than 50% in 30 days [29]. In addition, Zhao et al. found that miR-497 up-regulation in NSCLC cells significantly reduced xenograft tumor volume, weight and angiogenesis by targeting HDGF [16]. Moreover, when LV-miR-497-transfected Huh7 hepatoma cells were injected into the tail vein of nude mice, the number of metastatic lung nodules decreased markedly after four weeks compared with controls [67]. In prostate cancer, knocking down the expression of IKK β, a direct target of miR-497, can suppress cell proliferation, migration and invasion *in vitro*; this indicates that miR-497 targets also have therapeutic potential [95]. Notably, Li et al. directly injected an adenovirus-miR-497 sponge that could inhibit miR-497 expression into the left ventricular myocardium of mice 3 days before the onset of ischemia/reperfusion; they found a significant reduction in myocardial infarct size compared with controls [108].

miR-497 can also increase the sensitivity and response of tumor cells to drugs, and several reports suggest using it with chemotherapeutic agents. In osteosarcoma, Shao et al. found that cisplatin-induced cytotoxicity was markedly increased in SAOS-2 cells with enhanced miR-497 expression. Mice that received miR-497 and cisplatin combination therapy had smaller tumors than those given cisplatin alone, with reductions of more than 50% [72]. Consistently, enhanced miR-497 expression promoted tumor chemosensitivity to gemcitabine in PDAC [64], cisplatin in chemotherapy-resistant OC [59], 5-fluorouracil in CRC [36], and rituximab in DLBCL [41]. However, Lan et al. revealed that over-expressed miR-497 in glioma promoted chemotherapy resistance in glioma cells by targeting PDCD4 [40]. Therefore, appropriate treatment strategies should be chosen according to the specific functions of miR-497 in different cancers.

The intricate relationship between miR-497 and its downstream targets presents some risks when using miR-497 as a therapeutic tool. The most prominent risk is the serious adverse effects of off-target activity or altering miR-497 levels in normal, non-cancerous cells. There are also other issues with miR-497 use, such as the targeted delivery and stability of drugs.

## CONCLUSIONS

miR-497 is dysregulated in various cancers *via* a variety of mechanisms, including TFs, epigenetic alterations and genomic alterations. It acts as a key modulator of proliferation, differentiation, apoptosis, angiogenesis, migration and invasion. miR-497 functions mainly as a tumor suppressor gene. However, it also acts as an oncogene in different cancers by directly targeting various downstream genes and multiple signaling pathways. Increasing evidence indicates that miRNA-497 can serve as a diagnostic and prognostic biomarker and as a promising therapeutic target in future clinical application. Regardless, several issues require further investigation, such as the specificity for tumors as a diagnostic biomarker, adverse off-target effects, targeting infusion pathway and so on. There is a long way for application. Only a better understanding of miR-497 and its target genes will allow the successful translation of current research into clinical applications.
